# The Impact of Infections in Patients Treated with Atezolizumab Plus Bevacizumab for Unresectable Hepatocellular Carcinoma

**DOI:** 10.3390/jcm13174994

**Published:** 2024-08-23

**Authors:** Abdullah Esmail, Jiaqiong Xu, Ethan A. Burns, Karen Abboud, Ali Sheikh, Godsfavour Umoru, Kelly Gee, Catherine Wiechmann, Yuqi Zhang, Maen Abdelrahim

**Affiliations:** 1 Houston Methodist Neal Cancer Center, Houston Methodist Hospital, Houston, TX 77030, USA; 2 Department of Medicine, Houston Methodist Hospital, Houston, TX 77030, USA

**Keywords:** atezolizumab, bevacizumab, hepatocellular carcinoma, immune-related, adverse events, risk factors

## Abstract

**Background**: The therapeutic landscape of unresectable hepatocellular carcinoma (uHCC) continues to evolve. Atezolizumab, an anti-programmed cell death ligand 1 (PD-1) immune checkpoint inhibitor (ICI), in combination with bevacizumab, has substantially improved outcomes. This study aims to evaluate the incidence, risk factors, and outcomes in patients who develop infections while receiving atezolizumab and bevacizumab for uHCC. **Methods:** Patients who received atezolizumab and bevacizumab for uHCC at a single hospital network were included. Types and rates of infections were reported. Covariates compared among infected and non-infected cohorts included age, sex, race, comorbidities, Eastern Cooperative Oncology Group (ECOG) performance status, immunosuppressive use, chronic infections, number of cycles of ICIs given, antibiotic or antiviral therapies at ICI initiation, and line of therapy (first-line, second-line, greater than second-line). **Results**: Out of 810 evaluable patients, 34 uHCC patients were treated with atezolizumab plus bevacizumab. The mean ± SD age was 66.29 ± 9.39; 28 (82.35%) were males. There were 17 (50%) patients with reported infection, with bacterial infection occurring in 12 (70.59%) patients and COVID-19 in 4 (23.5%). Of the infected patients, eight (47.06%) had one infection, five (29.41%) had two infections, and two (11.76%) had three or more infections. Infected and non-infected patients received a median of 12 (IQR: 5–17) and 4 (IQR: 3–12) ICI cycles (*p* = 0.18), respectively. Infections did not negatively impact OS or PFS but resulted in treatment delays and discontinuation in 11 (64.71%) and 7 (41.18%) patients, respectively. At the last follow-up, 19 (55.88%) patients died, 9 (52.94%) in the non-infected group vs. 10 (58.82%) in the infected group (*p* = 1.0). **Conclusions**: While a broad array of infections occurred in 50% of the patients in this cohort, it did not negatively impact survival outcomes. However, it did impact morbidity, with more all-cause admissions and treatment delays.

## 1. Introduction

Unresectable hepatocellular carcinoma (uHCC) is the fifth most common cancer and the second leading cause of cancer-related deaths worldwide [1,2,3]. Recognized risk factors for HCC include male gender, age, obesity, years with cirrhosis, and family history of liver cancer [4]. Chronic liver disease and cirrhosis, the major risk factors for HCC, can arise from various causes including viral infections such as hepatitis B virus (HBV), hepatitis C virus (HCV), and/or HIV, type-2 diabetes leading to non-alcoholic fatty liver disease (NAFLD) and non-alcoholic steatohepatitis (NASH), or chronic alcohol consumption [5,6,7]. At the same time, cirrhosis undermines the immune system and predisposes patients to a wide range of infections, such as spontaneous bacterial peritonitis, spontaneous bacteremia, urinary tract infection, pneumonia, and skin and soft tissue infection [8,9,10,11].

Combining atezolizumab, an anti-programmed cell death ligand 1 (PD-L1) immune checkpoint inhibitor (ICI), with bevacizumab, an anti-vascular endothelial growth factor (anti-VEGF) monoclonal antibody, has improved survival outcomes. The combination leads to a synergistic effect by eliminating VEGF-induced immunosuppression and enabling T cell infiltration in the tumor microenvironment [12,13]. In the landmark phase III clinical trial IMbrave150, the 12-month overall survival (OS) rate for patients treated with atezolizumab and bevacizumab was 67.2% compared to 54.6% for patients who received sorafenib alone for uHCC [14]. A similar significant advantage was seen with the median progression-free survival (PFS), which was 6.8 months for the atezolizumab-bevacizumab group and 4.3 months for the sorafenib group. 

The infection risk associated with the use of atezolizumab and bevacizumab in HCC has not been investigated. A meta-analysis of 33,526 patients from 41 RCTs demonstrated a 1.45-fold higher risk of developing an infection of any grade and a 1.59-fold higher risk of high-grade infection with the use of bevacizumab. Of note, this meta-analysis did not include patients with HCC [15]. On the other hand, several retrospective studies have assessed the infection rate and impact in ICI-treated patients, which has varied substantially [16,17,18]. While immune-related adverse events (irAE) impacting virtually every organ have been thoroughly studied and reported, risk factors for infection, infection incidence, and complications arising from them in specific malignancies have not been studied. Infections are a common cause of treatment interruption, morbidity, and mortality in cancer patients, and whether ICIs harbor an added risk or lower risk needs to be further explored. 

However, in the broader terms of ICI treatment, certain known complications can occur due to therapy. Any specific causalities will scarcely be pinpointed this early on, so the question of the burden on participation is considered [19,20]. Regarding the infections and morbidity of the patients involved in specific ICI treatment studies, adherence to the protocols will strictly affect the reported mortality [21,22]. Since ICI has essentially transformed the intrinsic context of immunotherapy, this study aims to evaluate the incidence, risk factors, and outcomes in patients who develop infections while receiving atezolizumab and bevacizumab for HCC [23,24]. 

## 2. Methods

### 2.1. Study Design

This IRB-approved, observational, retrospective study assessed the incidence, risk factors, and outcomes in patients who developed infections while receiving atezolizumab and bevacizumab for uHCC. Data on individuals receiving atezolizumab at a single hospital network were reviewed. Patients ≥ 18 years of age at the time of diagnosis were included if they had a confirmed receipt of ≥1 cycle of atezolizumab either as a monotherapy or combined with bevacizumab within the study institution at the time of ICI initiation. Patients with missing data, missing follow-up information, or those lost to follow-up/received therapy elsewhere were excluded from the analysis.

### 2.2. Data Collection

Every patient included in the study underwent a chart review for inclusion criteria. The confirmation of infections was individually documented by credentialled physicians. An infection was documented if it was reported while actively on atezolizumab or within three weeks of treatment discontinuation. To be classified as an infection, the documentation of pertinent cultures, polymerase chain reactions, and antigen testing had to be confirmed by chart review in conjunction with the clinical picture documented. If no microbiological cultures were noted, clinical symptoms combined with pertinent radiographic features and infectious disease physician documentation were required. Because the primary objective of this study lies in evaluating the infection rate concerning survival outcomes, all types of infections were recorded. The number of infections per patient was classified as 1, 2, or ≥3, and the rate of the entire treatment population was reported. Comorbidities were defined as the presence of one or more additional medical conditions that co-occurred with the primary cancer. Total emergency department (ED), intensive care unit (ICU), and inpatient hospitalizations were recorded and compared between infected and non-infected cohorts. Furthermore, the effects of those incidents on the continuity of treatment, be they delays or discontinuation, were also documented. In addition, based on the primary objective, the deaths and disease progressions of patients in each cohort were recorded for further evaluation.

Covariates compared between infected and non-infected cohorts included age, sex (male/female), race (self-reported), comorbidities, Eastern Cooperative Oncology Group performance status (ECOG PS) (ranging from 0, defined as fully active, to 4, being completely disabled), immunosuppressive use with ICI use, history of solid or hematopoietic cell transplant, chronic infections, line of atezolizumab therapy administered (first-line (1L), second-line (2L), greater than second-line (>2L)), Rifaximin at ICI initiation, and antibiotic use at ICI initiation. 

The primary objective of this study was to evaluate the incidence of infections and the risk factors contributing to infection development in patients who received atezolizumab and bevacizumab for uHCC. Secondary objectives included morbidity (comparison of hospitalizations), impacts on treatment cycles received, and OS/PFS. 

### 2.3. Statistical Analysis and Outcomes

Baseline characteristics were presented as mean ± SD or median (IQR) for continuous variables and *n* (%) for categorical variables by infected vs. non-infected. Fisher’s exact test for categorical variables and Mann–Whitney test for continuous variables were used to compare patients with/without infection. Univariable logistic regression models were used to establish characteristics independently associated with infectious risk during ICI therapy. These outcomes were reported as odds ratio (OR), 95% confidence intervals (CI), and *p*-values. Kaplan–Meier analysis was implemented to compare OS and PFS between infected and non-infected cohorts. Univariable Cox proportional hazards regression was used to examine the association of baseline characteristics with OS and PFS. The results were reported as hazard ratios (HR) with 95% CI and *p*-values. The Cox proportionality assumption was verified by including the models’ time-dependent interactions of covariates with survival time. There was no violation of this assumption for any covariate. All analyses were performed with STATA version 17 (StataCorp. 2021. Stata Statistical Software: Release 17. College Station, TX, USA: StataCorp LLC.). Statistical significance was defined as two-tailed *p* < 0.05 for all tests.

## 3. Results

### 3.1. Baseline Factors 

Of the 810 patients with cancer who received atezolizumab, 34 of them had uHCC. Of these 34 patients, 17 (50%) had reported infections. Within this uHCC cohort, the mean age was 66.29 ± 9.39 years; 28 (82.35%) were males, 26 (76.47%) were white, and 20 (58.82%) had comorbidities. Of the patients who developed infection(s), eight (47.06%) had an ECOG PS of 0–1 while seven (41.18%) had an unknown ECOG status (Table 1).

### 3.2. Infections

Of the 34 evaluated patients, 17 (50%) had at least one infection, with 8 (47.06%) having one infection, 5 (29.41%) having two infections, 2 (11.76%) having three infections, and 2 (11.76%) having >3 infections. Among those, bacterial infections were the most common, occurring in 12 (70.59%) of 34 patients, while COVID-19 was reported in 4 (23.5%) of 34 patients (Table 1). Of 34 patients, 20 had at least one comorbidity, with 50% of the 20 patients falling under the infected subgroup. Of the 17 infected patients, 8 (47.06%) had a history of a previous chronic infection. Specifically, six (35.29%) patients had chronic hepatitis C (HCV), one (5.88%) patient had both chronic hepatitis B and HCV, and one (5.88%) patient had latent herpes simplex virus. Infected vs. non-infected subgroups received a median of 12 (5–17) vs. 4 (3–12) ICI cycles (*p* = 0.18). Of the 17 patients that developed infections, 17.65% were taking Rifaximin at the ICI initiation, whereas 29.51% were on antibiotics at ICI initiation. Though the retrospective number of ED and inpatient hospitalizations is not statistically significant, ICU admissions were significantly higher in the infected vs. non-infected group (0 (0%) vs. 5 (29.41%)) (*p* = 0.44). Moreover, although adverse events (AEs), immunosuppression, and local therapies were recorded, they did not affect a plurality of the patient population. 

The univariable analysis from logistic regression model results show that none of the baseline characteristics were significantly associated with infection (Table 2). 

### 3.3. Outcomes

**Figure 1:**
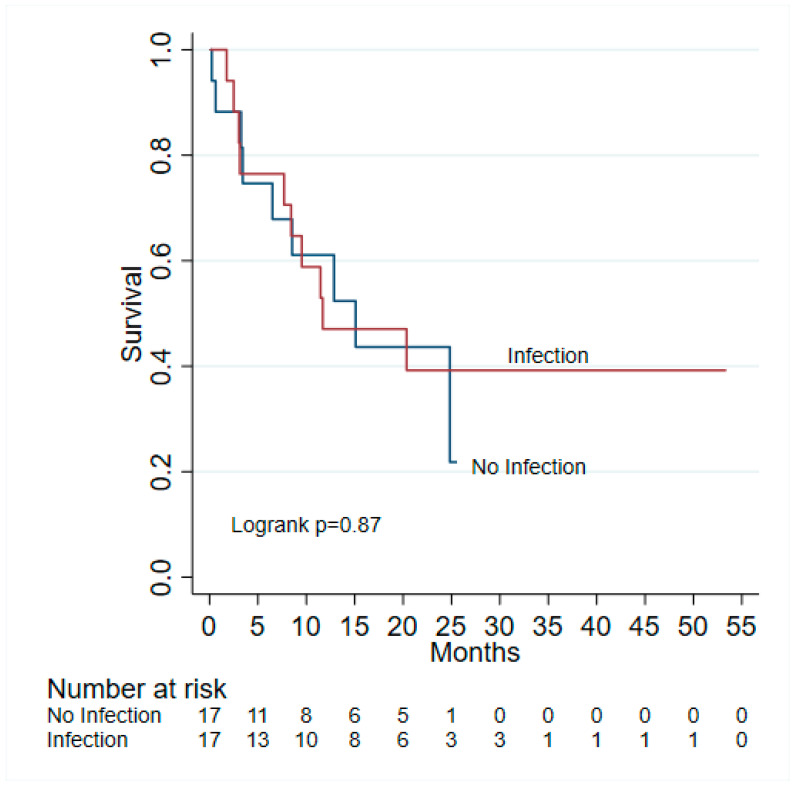
Kaplan–Meier analysis of overall survival.

**Figure 2:**
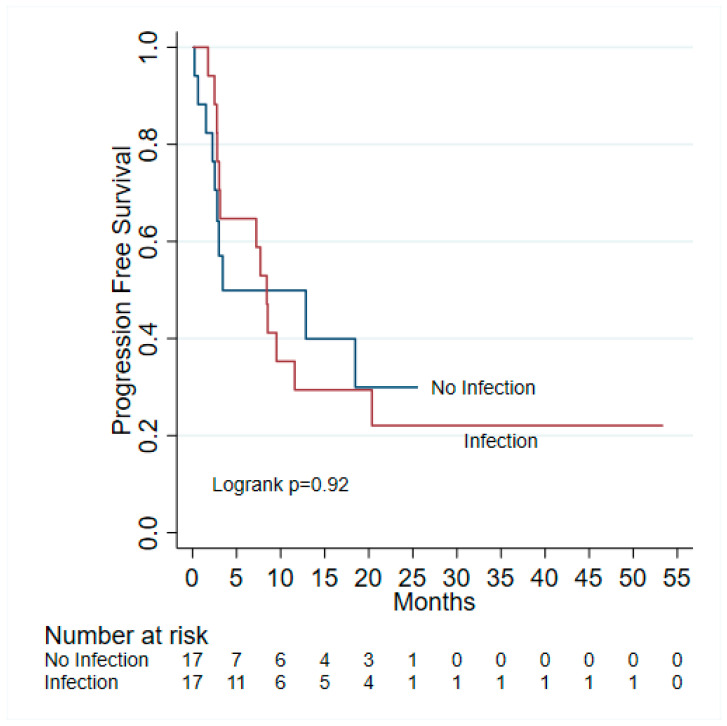
Kaplan–Meier analysis of progression-free survival.

A total of 10 (29.41%) of 34 patients experienced irAEs; of those, 6 of 17 (35.29%) occurred in the infected cohort and 4 of 17 (23.53%) in the non-infected cohort (*p* = 0.71) (Table 1). At the last follow-up, 19 (55.88%) of 34 patients had died, 9 (52.94%) of 17 patients from the non-infected cohort and 10 (58.82%) of 17 patients from the infected cohort (*p* = 1.0) (Table 1). The median follow-up time was 11.7 (IQR: 7.7–23.1) months and 8.57 (IQR: 3.27–20.03) months for infected and non-infected, respectively—there were no statistically significant differences in OS and PFS between infected and non-infected (Figure 1 and Figure 2). Of 17 patients, 11 (64.71%) had a delay or interruption in their treatment due to their infection, while 7 (41.18%) of 17 patients had their treatment discontinued as a result of their infection. Although infections were not statistically associated with either OS or PFS, some risk factors substantially impacted OS and PFS. Use of immunosuppressives (HR = 11.32, 95% CI 1.87–68.44, *p* = 0.008) and ICU admissions (HR = 3.67,95% CI 1.24–10.86, *p* = 0.019) were significantly associated with a higher risk of death. The number of ICI cycles was positively associated with OS (HR = 0.88, 95% CI 0.81–0.95, *p* = 0.002) and PFS (HR = 0.85, 95% CI 0.78–0.93, *p* < 0.001), whereas the number of ED visits was positively associated with PFS (HR = 0.30, 95% CI 0.10–0.90, *p* = 0.032) (Table 3). 

## 4. Discussion

Infections are the most common complications seen in cancer patients, with studies showing a significant associated morbidity and mortality [25,26]. The increased risk is due to a multitude of factors, including but not limited to the underlying malignancy and immunosuppression caused by the chemotherapy agents. However, the effect of immunotherapy on infection incidence and outcomes has not been very well studied in specific cancers. The landmark IMbrave150 trial changed the standard of care for uHCC, demonstrating atezolizumab and bevacizumab (atez/bev) to be superior to sorafenib alone. An updated analysis was presented in 2020, with results reporting more severe adverse events in those taking atez/bev compared to sorafenib [27]. However, they did not report specific data regarding the infection outcomes. To the best of our knowledge, this is the first study to report comprehensive infection outcomes in patients with uHCC who were treated with atez/bev. 

A meta-analysis conducted by Petrelli et al. reported the risk of infections as 9.6% for patients with cancer being treated with ICIs compared to 8.6% for patients receiving other treatment modalities [16]. In our study, the rate of infections was significantly higher, with half of the patients having at least one infection. This difference further highlights the variance in the infection incidence and outcomes that can occur due to the different types of cancers and treatments. A study conducted by Sang et al. analyzed the rate and outcomes of uHCC patients receiving atez/bev who were infected with COVID-19 only. They reported a COVID-19 infection rate of 14.5%, with a treatment delay occurring in 42.9% of those patients, with an additional 11.4% undergoing treatment discontinuation due to COVID-19 complications [28]. These figures are slightly lower than ours, with the likely explanations being differences in the profile of the infectious agents, patient population, and healthcare system differences. In a study of 102 patients undergoing systemic chemotherapy (either combination cisplatin, interferon, doxorubicin and fluorouracil (PIAF) or single-agent doxorubicin) for HCC, 58% of these patients developed hepatitis, of which 36% was caused by HBV reactivation. Notably, 30% of patients who experienced HBV reactivation died [29]. Now that the treatment paradigm has shifted away from systemic chemotherapy and towards immunotherapy, it is important to determine the rate of HBV reactivation, especially considering the high mortality rate associated with it. Fortunately, studies suggest that the risk in patients with HCC treated with ICIs is generally low, but is higher in HBsAg-positive patients compared to HBsAg-negative patients [30]. In one systematic review and meta-analysis looking at hepatitis B reactivation with ICIs for advanced cancer, the rate of reactivation was found to be 1.3%. However, a subgroup analysis showed that reactivation was more common in patients with HCC compared to other cancer types (1.9% (95% CI: 0–5.7%; I2 =  92.52%, *p*  <  0.001) vs. 0.5% (95% CI: 0–2.2%; I2  =  72.37%, *p*  <  0.001)). Another subgroup analysis showed that an HBsAg-positive status was associated with a higher risk of reactivation (1.3% (95% CI: 0–4.5%; I2 =  87.44%, *p*  <  0.001) and 0 (95% CI: 0–0; I2  =  0, *p*  =  0.796)) [31]. Other treatment options in uHCC include dual immunotherapy and tyrosine kinase inhibitors (TKIs) including regorafenib, cabozantinib, and ramucirumab [7,32]. Similar to bevacizumab, it can be extrapolated that these anti-VEGF inhibitors carry a small increase in the risk of infections, although this has not been evaluated in HCC [15]. 

Notably, while infected patients received fewer treatments, there was no difference in the median PFS or OS, possibly due to the small sample size. However, there was a higher number of ICU admissions for the infected patients subgroup, but no difference in overall hospitalization rates. Furthermore, pre-existing comorbidities did not confer an increased risk of developing infections. It is still uncertain how much co-morbidities contribute to the development of infections in patients treated with ICI. For example, a history of diabetes mellitus was an independent risk factor for infections in patients with lung cancer receiving nivolumab in the study by Fujita et al. On the other hand, diabetes did not show a significant association with infection risk in a retrospective chart review of solid tumor patients receiving ICIs [17].

In the sphere of research, it is becoming a generally known fact that infections can accompany any ICI immunotherapy treatments; notably, our study did not find an increased risk for infection in patients receiving immunosuppressive therapy at the initiation of atezolizumab or who received immunosuppression to manage irAE [33,34]. Sutthichai et al. evaluated the incidence of infection in patients on immunotherapy for solid tumors and hematologic malignancies [35]. They found that 68% of patients had de novo infections while on an ICI, and all these patients were started on immunosuppression for irAEs. The study suggested that some patients on ICI therapies, whether due to the ICI or specifically irAEs, will develop infections [14]. Several other recent studies found that immunosuppressive use, particularly systemic corticosteroids, predisposed patients to infections [35,36]. Conversely, like the present study, other findings suggest that factors aside from immunosuppressive use may drive the risk of infection development [37]. It has been posited that infections could develop due to a hyperinflammatory dysregulated immunity [37] or a hyper-responsive immunity similar to an immune reconstitution inflammatory syndrome (IRIS) that leads to the reactivation of chronic infections [38,39,40]. It could also be attributed to immunotherapy-induced lymphopenia. In addition, an increased risk of bacterial infections would translate to increased use of antibiotics. The use of antibiotics, which disrupts gut microbiota, has been previously associated with poorer outcomes in patients with HCC on ICI treatment; however, our study did not find any statistically significant detrimental effect on survival outcomes with antibiotic use [41]. Further research is needed regarding the relationship of antibiotic-mediated intestinal dysbiosis and response to immunotherapy, especially as fecal microbiota transplantation continues to be investigated for liver cirrhosis and treatment of immunotherapy-induced colitis [7,41,42,43]. 

Limitations of this study should be recognized. First, as in any retrospective chart review, there are natural biases that distinguish themselves from patient enrollment through study design and result outcomes. Along with the differences in baseline comorbidities, performance status, and treatment, the relatively small scale of the treatment group needs to give room for the better interpretation of the data. Furthermore, we did not have characteristic data on the treatment population for additional meta-analyses on the route of administration, type of chronic infection, or even distinguishing diverse population effects. Finally, given the retrospective nature of this chart review study, patients were excluded from the analysis if they did not have relevant inclusion criteria, as such as progress notes or imaging studies. This exclusion may have impacted the analysis of final results and skewed survival outcomes.

## 5. Conclusions

Infections were present in 50% of the patients receiving atezolizumab therapy for uHCC, showing that the incidence of infections during ICI therapy remains a common risk factor. It did not negatively impact OS or PFS. However, there was a general impact on morbidity for those who suffered from one or more infection(s) with an elevated rate of treatment delays and a significant rate of ICU admissions. More studies are needed to validate these outcomes.

## Figures and Tables

**Table 1:** Baseline characteristics by infection status.

	Total	No Infection	Infection	*p*-Value
	*N* = 34	*N* = 17	*N* = 17	
Age	66.29 ± 9.39	68.43 ± 10.47	64.15 ± 7.89	0.19
Sex				0.66
Male	28 (82.35)	13 (76.47)	15 (88.24)	
Female	6 (17.65)	4 (23.53)	2 (11.76)	
Race				0.69
White	26 (76.47)	12 (70.59)	14 (82.35)	
Other	8 (23.53)	5 (29.41)	3 (17.65)	
Comorbidities	20 (58.82)	10 (58.82)	10 (58.82)	1.00
ECOG PS				1.00
0	6 (17.65)	3 (17.65)	3 (17.65)	
1	11 (32.35)	6 (35.29)	5 (29.41)	
2	4 (11.76)	2 (11.76)	2 (11.76)	
Unknown	13 (38.24)	6 (35.29)	7 (41.18)	
History of Immunosuppressive Use	2 (5.88)	0 (0.00)	2 (11.76)	0.48
History of Solid or Hematopoietic Cell Transplant	3 (8.82)	1 (5.88)	2 (11.76)	1.00
Patients with Chronic Infection	21 (61.76)	13 (76.47)	8 (47.06)	0.16
Hepatitis B Only	1 (2.94)	1 (5.88)		
Hepatitis C Only	14 (41.17)	8 (47.06)	6 (35.29)	
Hepatitis B and C	3 (8.82)	2 (11.76)	1 (5.88)	
Hepatitis B and Latent TB	1 (2.94)	1 (5.88)		
Hepatitis B and C and Latent TB	1 (2.94)	1 (5.88)		
HSV	1 (2.94)		1 (5.88)	
Number of Cycles of ICIs Given	7 (3–13)	4 (3–12)	12 (5–17)	0.18
Line of Systemic Therapy				0.36
1	25 (73.53)	14 (82.35)	11 (64.71)	
2	6 (17.65)	3 (17.65)	3 (17.65)	
>2	3 (8.82)	0 (0.00)	3 (17.65)	
Rifaximin at ICI Initiation	4 (11.76)	1 (5.88)	3 (17.65)	0.60
Patients on Antibiotic	8 (23.53)	3 (17.65)	5 (29.41)	0.69
IrAE	10 (29.41)	4 (23.53)	6 (35.29)	0.71
Steroids for irAE	4 (28.57)	2 (33.33)	2 (25.00)	1.00
Number of ED Visits				0.64
0	24 (70.59)	13 (76.47)	11 (64.71)	
1	4 (11.76)	1 (5.88)	3 (17.65)	
2	5 (14.71)	3 (17.65)	2 (11.76)	
5	1 (2.94)	0 (0.00)	1 (5.88)	
Number of Inpatient Hospitalizations	1 (0–2)	1 (0–1)	1 (0–4)	0.098
ICU Admissions	5 (14.71)	0 (0.00)	5 (29.41)	0.044
Number of Reported Infections				
1			8 (47.06)	
2			5 (29.41)	
3			2 (11.76)	
>3			2 (11.76)	
Bacterial			12 (70.59)	
Infection Causing Treatment Delays/Interruptions			11 (64.71)	
Infection Resulting in Treatment Discontinuation			7 (41.18)	

Eastern Cooperative Oncology Group performance status (ECOG PS). TB: Tuberculosis. ICI: Immune checkpoint inhibitor. IrAE: Immune-related adverse events. ED: Emergency department. ICU: Intensive care unit.

**Table 2:** Risk factors for infection from a univariable logistic regression model.

	OR (95% CI)	*p*-Value
Age	1.06 (0.97–1.1)	0.178
Sex	5	
Male	Ref	
Female	0.43 (0.07, 2.76)	0.376
Race		
White	Ref	
Other	0.51 (0.10, 2.61)	0.423
Comorbidities	1.00 (0.26, 3.92)	1.000
ECOG PS		
0	Ref	
1	0.83 (0.11, 6.11)	0.858
2	1.00 (0.08, 12.56)	1.000
Unknown	1.17 (0.17, 8.09)	0.876
History of Immunosuppressive Use	NA	
History of Solid or Hematopoietic Cell Transplant	2.13 (0.17, 26.03)	0.553
Chronic Infection	0.27 (0.06, 1.19)	0.084
Number of Cycles ICIs Given	1.06 (0.97–1.15)	0.178
Line of Systemic Therapy		
1	Ref	
2	1.27 (0.21, 7.58)	0.791
>2	NA	
Rifaximin at ICI Initiation	3.43 (0.32, 36.83)	0.309
Was the Patient on Antibiotic	1.94 (0.38, 9.88)	0.423
IrAE	1.77 (0.40, 7.93)	0.454
Steroids for irAE	0.67 (0.06, 6.87)	0.733
ED Visits	1.77 (0.40, 7.93)	0.454
Inpatient Hospitalizations	2.13 (0.52, 8.76)	0.293
ICU Admissions	NA	

Eastern Cooperative Oncology Group performance status (ECOG PS). ICI: Immune checkpoint inhibitor. IrAE: Immune-related adverse events. ED: Emergency department. ICU: Intensive care unit. NA: Not Applicable.

**Table 3:** Association of risk factors with death and PFS.

	Death		PFS	
	HR (95% CI)	*p*-Value	HR (95% CI)	*p*-Value
Infection	0.93 (0.38–2.29)	0.873	1.04 (0.46, 2.39)	0.917
Age	1.04 (0.99–1.10)	0.097	1.02 (0.97–1.07)	0.460
Sex				
Male	Ref			
Female	0.28 (0.04, 2.10)	0.215	1.11 (0.33, 3.79)	0.864
Race				0.69
White	Ref			
Other	0.49 (0.14, 1.70)	0.261	0.29 (0.08, 1.00)	0.050
Comorbidities	1.31 (0.51, 3.35)	0.568	1.12 (0.48, 2.59)	0.795
ECOG PS				1.00
0	Ref		Ref	
1	0.79 (0.22–2.86)	0.725	0.39 (0.12, 1.33)	0.134
2	0.39 (0.04–3.52)	0.401	0.57 (0.11, 3.00)	0.506
5	0.90 (0.27–3.03)	0.868	0.77 (0.26, 2.30)	0.644
History of Immunosuppressive Use	11.32 (1.87, 68.44)	0.008 *	3.64 (0.79, 16.70)	0.097
History of Solid or Hematopoietic Cell Transplant	0.97 (0.22, 4.25)	0.969	0.67 (0.16, 2.91)	0.595
Chronic Infection	0.54 (0.22, 1.33)	0.178	0.52 (0.23, 1.20)	0.126
Number of ICI Cycles Given	0.88 (0.81–0.95)	0.002 *	0.85 (0.78–0.93)	<0.001 *
Line of Systemic Therapy				
1	Ref		Ref	
2	0.69 (0.20–2.43)	0.567	0.89 (0.30, 2.66)	0.838
>2	1.29 (0.29–5.74)	0.742	1.07 (0.25, 4.71)	0.924
Rifaximin at ICI Initiation	2.24 (0.64, 7.80)	0.204	1.32 (0.39, 4.45)	0.656
Antibiotic Use	1.13 (0.40, 3.18)	0.812	1.66 (0.68, 4.04)	0.267
IrAE	0.66 (0.23, 1.89)	0.438	0.69 (0.27, 1.76)	0.439
Steroids for irAE	0.63 (0.13, 3.06)	0.569	0.40 (0.08, 1.92)	0.253
ED Visits	0.50 (0.17, 1.53)	0.226	0.30 (0.10, 0.90)	0.032 *
Inpatient Hospitalizations	1.10 (0.42, 2.85)	0.852	0.54 (0.24, 1.24)	0.147
ICU Admissions	3.67 (1.24, 10.86)	0.019 *	1.79 (0.65, 4.95)	0.260
Death	19 (55.88)	9 (52.94)	10 (58.82)	1.0
Disease Progressed	10 (29.41)	5 (29.41)	5 (29.41)	1.0

Eastern Cooperative Oncology Group performance status (ECOG PS). ICI: Immune checkpoint inhibitor. IrAE: Immune-related adverse events. ED: Emergency department. ICU: Intensive care unit. * All font in bold highlight *p*-values with statistical significance.
